# Consumption of ultra- and non-ultra-processed foods of individuals with normal-weight obesity

**DOI:** 10.1017/jns.2023.51

**Published:** 2023-07-06

**Authors:** Acsa de Castro Santos, Anna Flavia Ferreira Passos, Luciana Bronzi de Souza, Alexandre Siqueira Guedes Coelho, Cristiane Cominetti

**Affiliations:** 1 Nutritional Genomics Research Group, Graduate Program in Nutrition and Health, School of Nutrition, Federal University of Goiás, Goiânia, Goiás, Brazil; 2 School of Nutrition, Federal University of Goiás, Goiânia, Goiás, Brazil; 3 Plant Genetics and Genomics Laboratory, School of Agronomy, Federal University of Goiás, Goiânia, GO, Brazil

**Keywords:** Adiposity, Adult, Dietary guideline, Food consumption, Lifestyle

## Abstract

The normal-weight obesity (NWO) is highly associated with an increased risk for chronic non-communicable diseases and intricately linked to diet quality. Therefore, we assessed the consumption of ultra-processed and non-ultraprocessed foods of 224 Brazilian adults with NWO (*n* 159) and without NWO (*n* 65, non-NWO) in a cross-sectional study. For that, three dietary recalls were applied and categorised according to the NOVA classification. Individuals with NWO showed lower energy intake from the ‘fresh or minimally processed food’ group, specifically for rice (*P* = 0⋅037), beans (*P* = 0⋅002) and fruits (*P* = 0⋅026), as well as lower consumption of dietary fibre (*P* < 0⋅05) compared with non-NWO subjects. Total consumption of ultra-processed foods did not differ between groups; however, individuals with NWO had a higher energy intake from processed meats compared with the non-NWO group (54⋅1 ± 73⋅5 × 32⋅5 ± 50⋅8 kcal; *P =* 0⋅023). Energy and added sugar from ultra-processed foods (OR 1⋅02, CI 95 % 1⋅00–1⋅04, *P* = 0⋅0100) and total fat from non-ultra-processed foods (OR 1⋅09, CI 95 % 1⋅01–1⋅18; *P* = 0⋅0100) were associated with the presence of NWO. In conclusion, non-NWO individuals consumed more non-ultra-processed foods compared with the NWO group. Overall, there were no differences in the consumption of ultra-processed foods between the two groups. Important associations between food compounds and the presence of NWO were observed, emphasising the importance of fresh and minimally processed foods as the basis of the diet.

## Introduction

Normal-weight obesity (NWO) is characterised by an adequate body mass index (BMI), and a high percentage of body fat (%BF), which results in an increased risk of developing nutrition-related non-communicable diseases (N-NCDs)^(1)^. In addition to being a primary risk factor strongly associated with N-NCDs, high %BF impairs physical function and reduces quality of life^(2)^. Epidemiological studies highlight the importance of dietary habits in determining body composition, which together with physical activity and genetics, play a fundamental role in the modifiable causes of N-NCDs^(3,4)^.

There are multiple factors contributing to changes in current dietary patterns, including the rising production and consumption of industrially processed foods and beverages^(5)^. Changes in food access and quality have been influenced by shifts in production and distribution, as well as social, cultural and economic changes^(6)^. These changes have led to a significant increase in the prevalence of overweight and obesity in Brazil^(7)^.

Considering the negative changes in the diet quality of the Brazilian population in the last decades^(8)^, healthy dietary habits are being encouraged through clear and accessible information from the national dietary guidelines^(9)^. Based on the information that several dietary guidelines have neglected the impact of industrial processing on overall health^(10,11)^, a new classification of foods (NOVA) clustering them according to the extent and purpose of their processing was proposed^(11)^. In this classification, processing is considered the physical, chemical and biological procedures following the harvest of raw food and before it is subjected to any culinary preparation^(11)^. This new classification was adopted as a theoretical framework for the current Dietary Guidelines for the Brazilian population^(9)^.

Therefore, it is important to identify factors related to the diet of individuals with NWO, as they may present food consumption that favours the excessive accumulation of body fat. Here, we aimed to describe the consumption of ultra- and non-ultra-processed foods by individuals with and without NWO, as well as investigate the association of food and nutrient intake to the presence of NWO. We hypothesised that individuals with NWO have a higher consumption of ultra-processed foods compared to their counterparts and that there are associations between the intake of this type of food and the NWO.

## Material and methods

### Study design, participants and ethics

**Fig. 1.**
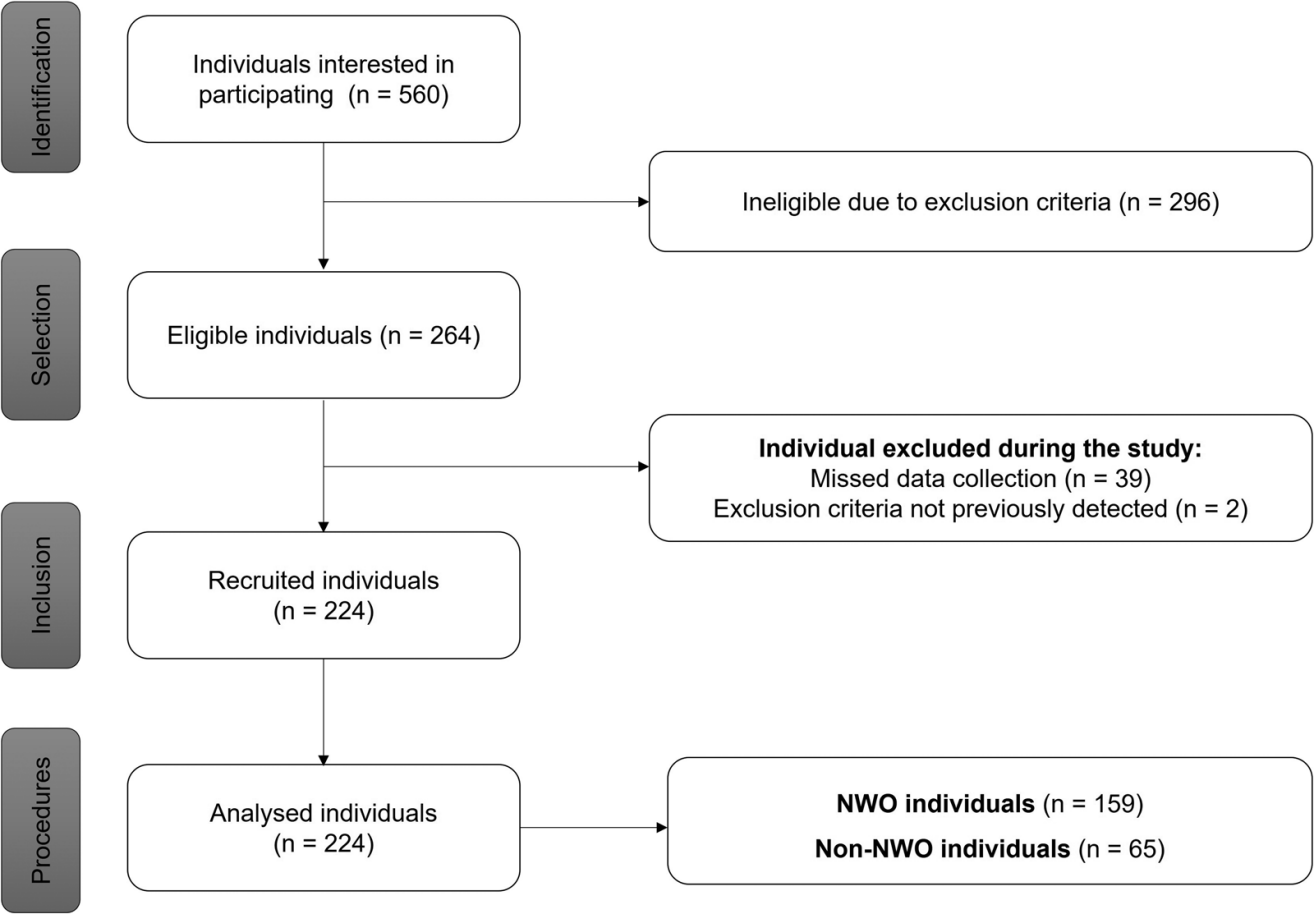
STROBE flowchart of participants’ recruitment.

This was a cross-sectional study carried out in an academic community in Goiânia, Goiás, Brazil, from January to June 2019. The study was publicised through folders, social networks and e-mails sent to students, employees and professors at the Federal University of Goiás (UFG). Individuals aged between 20 and 59 years and with normal BMI (18⋅50–24⋅99 kg/m²)^(12)^ were eligible for the study. Exclusion criteria were smoking, use of metal implants, limb amputation, intense physical activity, self-reported vitamin and/or mineral supplementation, self-reported acute and/or chronic diseases, self-reported use of lipid-lowering drugs, anti-hypertensives, anti-glycaemic drugs or insulin; pregnant or lactating women; menopause or undergoing hormone replacement therapy; undergoing nutritional monitoring and/or changing usual diet in the last 6 months prior to collection; or missing some stage of data collection. The final sample size consisted of 159 NWO individuals and 65 non-NWO individuals (Fig. 1).

This study was conducted according to the guidelines laid down in the Declaration of Helsinki and all procedures involving human subjects were approved by the Ethics Committee of the Federal University of Goiás (protocol number 2.772.022). Written informed consent was obtained from all subjects.

### Data collection

Data were collected at the School of Nutrition of the Federal University of Goiás, considering the availability of the participants. During the first appointment, participants signed the consent form, answered a standardised questionnaire (including information about food consumption), and had the body composition assessed. At the second appointment, participants were contacted by phone calls to provide additional information on food consumption.

Lifestyle assessment included routine habits, such as alcohol consumption and level of physical activity, which was assessed with the short version of the International Physical Activity Questionnaire^(13)^. The classification included very active, active, irregularly active (A or B) and sedentary.

### Body composition assessment

Body composition (percentage of total BF, android BF and gynoid BF) was evaluated using Dual Energy Radiological Absorption (DXA) with a DPX-NT model equipment (General Electric Medical Systems Lunar^®^, Madison, USA)^(14)^.

### Anthropometry

Body mass was measured on a Filizola^®^ digital platform scale, 150 kg maximum load and 0⋅1 kg accuracy (Filizola Shop, São Paulo, Brazil). Height was assessed using a Seca^®^ stadiometer, 220 cm maximum range and 0⋅1 cm accuracy (Seca Deutschland, Hamburg, Germany)^(15)^.

### NWO classification cut-off points

Individuals with normal BMI (18⋅50–24⋅99 kg/m^2^) were separated by sex and age group and classified according to their %BF determined by DXA. The groups were women with NWO – %BF >30 %^(1)^ and men with NWO – %BF >19 %^(16)^.

### Food consumption analysis

The food consumption was evaluated using three 24 h recalls (R24h) on non-consecutive days and including a weekend day^(17)^, following the Multiple Pass Method (MPM)^(18)^. To mitigate collection bias, during face-to-face and telephone interactions, trained researchers used strategies to quantify the food servings, such as a photographic manual and household measuring utensils^(19)^. Then, this information was converted from household measures to grams^(20)^ and the data were analysed using the Nutrition Data System for Research (NDS-R^®^) software (Nutrition Coordinating Center, University of Minnesota, Minneapolis – Saint Paul, USA)^(21)^. Once the food consumption database was generated, the researchers established food standardizations, especially for those considered to be regional, since the NDS-R^®^ provides information from international food composition tables.

### The NOVA food classification

Foodstuffs were categorised by a registered dietitian (RD) according to the methodology proposed by Monteiro *et al.*^(22,23)^. Foods were classified into four groups according to the extension and purpose of the processing during production. Processing was defined as the methods and techniques used by the food and beverage industry to modify and convert them into food products, as well as the methods used to preserve and increase shelf life. Procedures performed during culinary preparation were not considered in the classification. After food categorisation, another RD inspected the entire list of foods to ensure the reliability of the categories. To classify the list of foods, RDs remained blind regarding the classification of individuals with and without NWO.

#### Group 1: fresh or minimally processed foods

The first group included those foods that have undergone minimal modification to increase shelf life and allow longer storage. Another purpose of minimal processing is to facilitate or diversify the culinary preparation of food, which include cleaning, portioning and removing non-edible parts. Other processes include grating, peeling, compression, simple bottling, drying, cooling, freezing, pasteurisation, fermentation, fat reduction, vacuum, gas and simple packaging^(22,23)^.

#### Group 2: culinary ingredients

The second group included substances extracted from group 1 or from nature that are consumed as necessary ingredients for culinary preparation. The main processes include pressing, grinding, spraying, drying and refining. The purpose of this processing is to provide products to be used in residential kitchens and restaurants to season, prepare or preserve foods from the first group. Rarely, ingredients in this category will be consumed in the absence of foods from group 1^(22,23)^.

#### Group 3: processed foods

The third group included foods prepared by adding salt or sugar and, occasionally, oil, vinegar or another ingredient from the second group to a food from the first group. These processes include preservation, cooking and non-alcoholic fermentation. In this category, the primary objective is to increase the food shelf life or modify its flavour^(22,23)^.

#### Group 4: ultra-processed foods

This group consists of industrial formulations produced with five or more ingredients. These ingredients mostly include substances and additives added during the manufacturing of processed foods. These substances are not common in culinary preparations as their purpose is to simulate sensory attributes of foods from the first group. The main objective of ultra-processing is to produce industrial ready-to-eat products to minimise preparation and replace foods from the first and third groups^(22,23)^.

### Statistical analyses and justification of sample size

Double-entry databases were built to check consistency and the analysis was performed using R software version 4⋅1⋅1^(24)^. First, the energy contribution of each NOVA classification group and subgroup was calculated. The proportion of energy of each group or subgroup in relation to total energy consumption was then calculated separately for every individual, and for the NWO and non-NWO individuals.

Total diet was divided into two dietary fractions: (1) ultra-processed foods and (2) non-ultra-processed foods (represented by fresh or minimally processed foods, culinary ingredients and processed foods). Nutrient intake was calculated for (1) total diet of NWO and non-NWO individuals; (2) ultra-processed and non-ultra-processed foods of the total sample; (3) ultra-processed foods of NWO and non-NWO individuals and (4) non-ultra-processed foods of NWO and non-NWO individuals.

Data distribution was assessed using the Shapiro–Wilk test. NWO and non-NWO individuals and the ultra- and non-ultra-processed fractions were compared using a Student's *t* test or Mann–Whitney test, according to the data distribution. Comparison of frequencies was performed using the χ^2^ test or Fisher's exact tests.

Logistic linear regression models were built to analyse the associations between the presence of NWO and food intake variables using the backward strategy for adjustment. Nutrients from ultra- and non-ultra-processed foods that could associate with NWO included energy, protein, carbohydrate, total and added sugars, total fat, alcohol, saturated fatty acids, trans fatty acids, cholesterol, fibre, calcium, iron and sodium.

Considering the lack of population-based studies with individuals with NWO in Brazil, the detectable effect size of the sample was settled *a posteriori*. Our sample size had a power of 80 % to detect, at a significance level of 5 %, a Cohen's effect size of magnitude 0⋅41. A significance level of 5 % was considered for all analyses.

## Results

The final sample consisted of 224 individuals with normal BMI, 159 (71⋅0 %) with high %BF (NWO group) and 65 with normal %BF (non-NWO group). Most subjects were female in both groups (*P* = 0⋅376). Furthermore, the groups did not differ with respect to marital status (*P* = 0⋅455), socioeconomic classification (*P* = 0⋅094), age (*P* = 0⋅506), height (*P* = 0⋅744) or physical activity level (*P* = 0⋅295). Individuals with NWO had higher levels of education (*P* = 0⋅046), weight (*P* = 0⋅019), BMI (*P* = <0⋅0001) and %BF (*P* = <0⋅0001). All descriptive data are presented in Table 1.

**Table 1.** Socioeconomic, lifestyle, anthropometric and body composition variables of individuals in the NWO and non-NWO groups

	Total (*n* 224–100 %)	NWO (*n* 159–71 %)	Non-NWO (*n* 65–29 %)	*P*-value*
Sex				0⋅376
Male	73 (32⋅6)	49 (30⋅8)	24 (36⋅9)	
Female	151 (67⋅4)	110 (69⋅2)	41 (63⋅1)	
Marital status				0⋅455
Single	200 (89⋅3)	139 (87⋅4)	61 (93⋅8)	
Married	23 (10⋅3)	19 (11⋅9)	4 (6⋅2)	
Divorced	1 (0⋅4)	1 (0⋅7)	0 (0)	
Education level				**0⋅046**
Incomplete under graduation	167 (74⋅6)	114 (71⋅7)	53 (81⋅5)	
Under graduated	45 (29⋅1)	33 (20⋅7)	12 (18⋅5)	
Graduated	12 (5⋅4)	12 (7⋅6)	0 (0)	
Economic classification				0⋅094
Higher	144 (64⋅3)	107 (63⋅3)	37 (56⋅9)	
Intermediate	79 (35⋅3)	52 (32⋅7)	27 (41⋅5)	
Lower	0 (0)	0 (0)	1 (1⋅5)	
Age (years)	23⋅0 (21⋅0–25⋅0)	23⋅0 (21⋅0–26⋅0)	23⋅0 (21⋅0–24⋅0)	0⋅506
Weight (kg)	60⋅7 (55⋅0–66⋅6)	61⋅3 (56⋅0–66⋅7)	57⋅4 (53⋅1–65⋅9)	**0⋅027**
Height (m)	1⋅7 (1⋅6–1⋅7)	1⋅7 (1⋅6–1⋅7)	1⋅7 (1⋅6–1⋅7)	0⋅696
BMI (kg/m²)	21⋅8 ± 1⋅5	22⋅1 ± 1⋅5	21⋅0 ± 1⋅4	**<0⋅0001**
PAL (ME in min/week)	260⋅0 (130⋅0–409⋅2)	240⋅0 (100⋅0–407⋅5)	307⋅6 ± 212⋅4	0⋅295
%BF DEXA	30⋅4 (23⋅4–36⋅5)	34⋅2 (28⋅8–37⋅8)	24⋅6 (15⋅4–28⋅1)	**<0⋅0001**
% BF Android	30⋅8 ± 9⋅7	34⋅8 ± 7⋅6	20⋅9 ± 6⋅4	**<0⋅0001**
% BF Gynoid	42⋅9 (34⋅2–48⋅4)	46⋅5 (38⋅1–49⋅6)	36⋅8 (23⋅2–41⋅0)	**<0⋅0001**
A/G	0⋅76 ± 0⋅15	0⋅80 ± 0⋅13	0⋅67 ± 0⋅16	**<0⋅0001**

Values are presented as mean ± sd, median (IQR) or absolute (relative) frequencies.

NWO, normal-weight obesity; BMI, body mass index; PAL, physical activity level; ME, metabolic equivalents; %BF DEXA, percentage of body fat assessed by Dual Energy Radiological Absorption; % BF Android, percentage of android body fat; % BF Gynoid, percentage of gynoid body fat; A/G, ratio between android/gynoid body fat.

Bold values indicate significant differences between groups.

* Student's *t* test or Mann–Whitney test, χ^2^ test or Fisher's exact test.

For NOVA classification, 49⋅4 % of the energy came from ‘Fresh or minimally processed foods’, 10⋅6 % from ‘Fats and Culinary Ingredients’, 12⋅8 % from ‘Processed foods’ and 27⋅2 % from ‘Ultra-processed foods’ (Table 2).

**Table 2.** Absolute and relative mean daily energy intake according to the NOVA food groups for the total sample (*n* 224)

NOVA food groups*	Order	kcal	%
Fresh or minimally processed foods	1	1083⋅6 ± 429⋅7	49⋅9 ± 12⋅9
Rice	1	130⋅4 ± 105⋅0	6⋅0 ± 4⋅3
Beans	2	81⋅3 ± 79⋅6	3⋅9 ± 3⋅9
Other legumes	3	2⋅8 ± 11⋅1	0⋅14 ± 0⋅55
Beef	4	254⋅2 ± 237⋅9	11⋅3 ± 9⋅2
Pork	5	57⋅1 ± 100⋅0	2⋅6 ± 4⋅1
Fruits	6	92⋅9 ± 92⋅0	4⋅4 ± 4⋅2
Other cereals	7	12⋅2 ± 22⋅8	0⋅66 ± 1⋅5
Milk	8	80⋅5 ± 83⋅4	3⋅8 ± 4⋅0
Poultry meat	9	98⋅3 ± 118⋅0	4⋅5 ± 5⋅0
Roots and tubers	10	69⋅4 ± 103⋅7	3⋅1 ± 4⋅1
Coffee and teas	11	1⋅7 ± 4⋅5	0⋅08 ± 0⋅19
Fishes	12	14⋅4 ± 45⋅1	0⋅72 ± 2⋅3
Leafy vegetables	13	6⋅6 ± 7⋅4	0⋅32 ± 0⋅35
Other vegetables	14	15⋅1 ± 20⋅9	0⋅73 ± 0⋅85
Eggs	15	5⋅9 ± 16⋅8	0⋅30 ± 0⋅87
Other fresh or minimally processed foods	16	106⋅4 ± 144⋅7	4⋅8 ± 6⋅1
Fats and culinary ingredients	2	233⋅9 ± 145⋅1	10⋅8 ± 5⋅8
Animal	17	38⋅9 ± 60⋅7	1⋅8 ± 2⋅5
Vegetable	18	137⋅1 ± 92⋅7	6⋅3 ± 3⋅8
Margarine	19	18⋅9 ± 78⋅0	0⋅89 ± 3⋅3
Sugar	20	58⋅4 ± 80⋅7	2⋅7 ± 3⋅5
Other culinary ingredients	21	173⋅2 ± 148⋅2	7⋅7 ± 5⋅5
Processed food	3	280⋅6 ± 207⋅8	12⋅6 ± 7⋅7
French bread	22	52⋅7 ± 87⋅9	2⋅4 ± 3⋅8
Cheeses	23	93⋅1 ± 100⋅4	4⋅2 ± 4⋅3
Canned or preserved meats	24	0⋅6 ± 6⋅8	0⋅03 ± 0⋅36
Canned vegetables	25	0⋅9 ± 3⋅7	0⋅05 ± 0⋅23
Other processed foods	37	49⋅9 ± 80⋅8	2⋅3 ± 3⋅6
Ultra-processed food	4	597⋅1 ± 355⋅3	26⋅8 ± 12⋅1
Sweet cakes, pies and cookies	26	63⋅8 ± 112⋅8	2⋅8 ± 4⋅6
Industrialised soft drinks and juices	27	94⋅8 ± 90⋅6	4⋅3 ± 3⋅9
Bread: loaf, hamburger, hot dog and similar	29	83⋅7 ± 93⋅1	3⋅8 ± 4⋅2
Candies	30	78⋅7 ± 118⋅3	3⋅4 ± 4⋅9
Crackers, savoury snacks (snacks)	31	50⋅3 ± 116⋅9	2⋅2 ± 4⋅6
Processed meats	32	47⋅9 ± 68⋅3	2⋅3 ± 3⋅4
Ready-made or semi-prepared dishes	33	8⋅0 ± 33⋅6	0⋅33 ± 1⋅3
Sweetened dairy drinks	34	17⋅3 ± 41⋅8	0⋅83 ± 2⋅0
Other ultra-processed foods	35	70⋅9 ± 102⋅4	3⋅2 ± 4⋅1
Alcoholic beverages	36	26⋅9 ± 113⋅3	1⋅0 ± 3⋅8
**TOTAL**		2195⋅2 ± 714⋅3	100⋅0 ± 0⋅00

Values are mean ± sd.

%, percentage in relation to the total energy intake.

* Adapted from (22,23).

When individuals were separated into groups, those in the NWO group exhibited overall lower energy intake (*P* = 0⋅030), but also lower mean energy from ‘fresh or minimally processed foods’, specifically for rice (*P* = 0⋅037), beans (*P* = 0⋅002) and fruits (*P* = 0⋅026) when compared with those in the non-NWO group. There were no differences in energy intake from ‘fats and culinary ingredients’ (*P* = 0⋅727), ‘processed foods’ (*P* = 0⋅345) and ‘ultra-processed foods’ (*P* = 0⋅874) between the two groups. Although the total energy intake from ultra-processed foods did not differ between the groups, there was a difference in energy intake from savoury crackers and snacks (*P* = 0⋅010), which was higher in the non-NWO group; and from sausages (*P* = 0⋅023), which was higher in the NWO group (Table 3).

**Table 3.** Total energy intake according to the NOVA food groups of individuals in the NWO (*n* 159) and non-NWO (*n* 65) groups

Order	NOVA food groups*	NWO	Non-NWO		NWO	Non-NWO	
		kcal		*P-*value*	%		*P-*value*
1	Fresh or minimally processed foods	1025⋅1 ± 390⋅9	1226⋅8 ± 486⋅6	**0⋅004**	48⋅9 ± 12⋅9	52⋅3 ± 12⋅8	0⋅069
1	Rice	124⋅4 ± 105⋅2	145⋅0 ± 103⋅9	**0⋅037**	5⋅8 ± 4⋅4	6⋅4 ± 4⋅0	0⋅195
2	Beans	70⋅7 ± 71⋅1	107⋅1 ± 92⋅9	**0⋅002**	3⋅5 ± 3⋅8	4⋅7 ± 4⋅0	**0⋅008**
3	Other legumes	1⋅3 ± 4⋅6	6⋅4 ± 19⋅0	0⋅501	0⋅07 ± 0⋅29	0⋅30 ± 0⋅90	0⋅509
4	Beef	251⋅4 ± 233⋅8	261⋅0 ± 249⋅4	0⋅655	11⋅6 ± 9⋅5	10⋅8 ± 8⋅2	0⋅878
5	Pork	61⋅0 ± 102⋅1	47⋅7 ± 94⋅5	0⋅383	2⋅8 ± 4⋅2	2⋅1 ± 4⋅0	0⋅288
6	Fruits	84⋅4 ± 83⋅1	113⋅6 ± 108⋅8	**0⋅026**	4⋅2 ± 4⋅2	4⋅9 ± 4⋅0	0⋅068
7	Other cereals	13⋅4 ± 24⋅2	9⋅3 ± 18⋅7	0⋅089	0⋅75 ± 1⋅6	0⋅40 ± 1⋅0	0⋅066
8	Milk	79⋅7 ± 84⋅2	82⋅5 ± 82⋅1	0⋅649	3⋅9 ± 4⋅1	3⋅6 ± 3⋅8	0⋅635
9	Poultry meat	96⋅0 ± 115⋅0	103⋅8 ± 125⋅6	0⋅660	4⋅5 ± 5⋅2	4⋅3 ± 4⋅4	0⋅877
10	Roots and tubers	63⋅6 ± 73⋅6	83⋅6 ± 154⋅2	0⋅770	3⋅0 ± 3⋅4	3⋅3 ± 5⋅4	0⋅584
11	Coffee and teas	1⋅6 ± 4⋅0	2⋅1 ± 5⋅5	0⋅052	0⋅08 ± 0⋅16	0⋅09 ± 0⋅25	0⋅181
12	Fishes	10⋅6 ± 37⋅6	23⋅8 ± 58⋅9	0⋅204	0⋅53 ± 1⋅9	1⋅2 ± 3⋅1	0⋅225
13	Leafy vegetables	6⋅2 ± 7⋅0	7⋅5 ± 8⋅1	0⋅293	0⋅32 ± 0⋅36	0⋅33 ± 0⋅33	0⋅499
14	Other vegetables	15⋅0 ± 23⋅5	15⋅3 ± 12⋅3	0⋅441	0⋅74 ± 0⋅94	0⋅70 ± 0⋅58	0⋅986
15	Eggs	6⋅2 ± 16⋅5	5⋅3 ± 17⋅7	0⋅178	0⋅31 ± 0⋅84	0⋅26 ± 0⋅94	0⋅154
16	Other fresh or minimally processed foods	90⋅1 ± 114⋅7	146⋅2 ± 195⋅4	**0⋅022**	4⋅2 ± 5⋅4	6⋅2 ± 7⋅3	0⋅024
2	Fats and culinary ingredients	237⋅9 ± 158⋅0	224⋅2 ± 107⋅9	0⋅727	11⋅2 ± 6⋅4	9⋅8 ± 4⋅2	0⋅348
17	Animal	39⋅0 ± 57⋅9	38⋅8 ± 67⋅4	0⋅590	1⋅8 ± 2⋅6	1⋅7 ± 2⋅5	0⋅499
18	Vegetable	139⋅6 ± 98⋅3	131⋅0 ± 77⋅7	0⋅986	6⋅6 ± 4⋅1	5⋅6 ± 2⋅7	0⋅239
19	Margarine	13⋅9 ± 34⋅2	30⋅9 ± 134⋅6	0⋅262	0⋅72 ± 1⋅7	1⋅3 ± 5⋅4	0⋅346
20	Sugar	59⋅9 ± 86⋅3	54⋅7 ± 65⋅5	0⋅767	2⋅8 ± 3⋅7	2⋅5 ± 3⋅1	0⋅960
21	Other culinary ingredients	168⋅7 ± 148⋅7	184⋅1 ± 147⋅6	0⋅394	7⋅9 ± 5⋅7	7⋅5 ± 5⋅0	0⋅958
3	Processed foods	270⋅4 ± 198⋅6	305⋅5 ± 228⋅5	0⋅345	12⋅4 ± 7⋅4	12⋅9 ± 8⋅4	0⋅778
22	French bread	46⋅6 ± 76⋅1	67⋅8 ± 111⋅0	0⋅415	2⋅2 ± 3⋅5	2⋅9 ± 4⋅3	0⋅546
23	Cheeses	91⋅1 ± 97⋅3	98⋅0 ± 108⋅2	0⋅926	4⋅2 ± 4⋅2	4⋅3 ± 4⋅6	0⋅733
24	Canned or preserved meats	0⋅24 ± 3⋅0	1⋅4 ± 11⋅7	0⋅512	0⋅01 ± 0⋅14	0⋅08 ± 0⋅63	0⋅512
25	Canned vegetables	0⋅98 ± 4⋅4	0⋅57 ± 1⋅2	0⋅242	0⋅05 ± 0⋅28	0⋅02 ± 0⋅05	0⋅184
37	Other processed foods	54⋅9 ± 87⋅5	37⋅9 ± 60⋅3	0⋅080	2⋅5 ± 3⋅9	1⋅7 ± 2⋅8	0⋅062
4	Ultra-processed foods	599⋅1 ± 357⋅4	592⋅3 ± 353⋅0	0⋅874	27⋅5 ± 12⋅3	24⋅9 ± 11⋅6	0⋅147
26	Sweet cakes, pies and cookies	66⋅6 ± 125⋅8	57⋅1 ± 72⋅0	0⋅472	2⋅9 ± 5⋅0	2⋅6 ± 3⋅5	0⋅535
27	Industrialised soft drinks and juices	91⋅8 ± 83⋅5	102⋅1 ± 106⋅2	0⋅926	4⋅3 ± 3⋅9	4⋅2 ± 4⋅1	0⋅679
29	Bread: loaf, hamburger, hot dog and similar	85⋅5 ± 91⋅9	79⋅2 ± 96⋅6	0⋅401	4⋅1 ± 4⋅5	3⋅2 ± 3⋅5	0⋅247
30	Candies	77⋅9 ± 124⋅1	80⋅9 ± 103⋅5	0⋅421	3⋅5 ± 5⋅1	3⋅3 ± 4⋅2	0⋅560
31	Crackers, savoury snacks (snacks)	40⋅1 ± 107⋅9	75⋅2 ± 134⋅0	**0⋅010**	1⋅8 ± 4⋅4	3⋅1 ± 5⋅1	**0⋅009**
32	Processed meats	54⋅1 ± 73⋅5	32⋅5 ± 50⋅8	**0⋅023**	2⋅7 ± 3⋅8	1⋅5 ± 2⋅2	**0⋅014**
33	Ready-made or semi-prepared dishes	9⋅3 ± 36⋅7	4⋅7 ± 24⋅3	0⋅906	0⋅38 ± 1⋅4	0⋅19 ± 0⋅92	0⋅901
34	Sweetened dairy drinks	18⋅4 ± 43⋅6	14⋅5 ± 37⋅2	0⋅259	0⋅89 ± 2⋅1	0⋅67 ± 1⋅8	0⋅240
35	Other ultra-processed foods	69⋅8 ± 102⋅2	73⋅8 ± 103⋅6	0⋅721	3⋅3 ± 4⋅3	2⋅9 ± 3⋅5	0⋅418
36	Alcoholic Beverages	28⋅4 ± 126⋅5	23⋅4 ± 72⋅4	0⋅896	1⋅1 ± 4⋅1	0⋅93 ± 2⋅8	0⋅878
Total		2132⋅4 ± 705⋅3	2348⋅8 ± 718⋅5	**0⋅030**	100⋅0	100⋅0	-

Values are mean ± sd.

%, percentage in relation to the total energy intake.

Student's *t* test or Wilcoxon test.

NWO, normal-weight obesity.

Bold values indicate significant differences between groups.

* Adapted from (22,23).

Next, we evaluated selected nutrients in relation to the overall diet. Differences between NWO and non-NWO individuals were found only for dietary fibre intake in which higher values were observed for the non-NWO group. When NWO and non-NWO groups were compared in relation to the ultra-processed food group no differences were observed for any nutrient. However, in the non-ultra-processed food group, NWO individuals exhibited lower consumption of energy, protein, carbohydrates, dietary fibre, calcium, iron, sodium and sugar compared with the non-NWO subjects (Table 4).

**Table 4.** Nutrient content of the overall diet and two NOVA groups of individuals in the NWO (*n* 159) and non-NWO (*n* 65) groups

Nutrients	Overall diet		Overall food groups (*n* 224)		UP food group		Non-UP food group	
	NWO	Non-NWO	UP	Non-UP	NWO	Non-NWO	NWO	Non-NWO
Energy	2132⋅4 ± 705⋅3	2348⋅8 ± 718⋅5*	597⋅1 ± 355⋅3	532⋅7 ± 187⋅2^¶^	599⋅1 ± 357⋅4	592⋅3 ± 353⋅0	511⋅1 ± 176⋅9	585⋅5 ± 202⋅2^†^
Protein	91⋅0 ± 34⋅8	100⋅8 ± 37⋅3	13⋅8 ± 10⋅4	26⋅7 ± 11⋅1^¶^	14⋅4 ± 11⋅1	12⋅1 ± 8⋅2	25⋅5 ± 10⋅6	29⋅6 ± 11⋅8^†^
Carbohydrate	244⋅6 ± 85⋅6	281⋅5 ± 97⋅6	81⋅1 ± 53⋅0	58⋅1 ± 23⋅1^¶^	80⋅3 ± 53⋅8	83⋅0 ± 51⋅5	54⋅8 ± 21⋅1	66⋅2 ± 25⋅7^†^
Total sugars	96⋅8 ± 47⋅2	101⋅9 ± 42⋅6	48⋅9 ± 36⋅1	16⋅5 ± 9⋅3^¶^	49⋅3 ± 38⋅2	47⋅9 ± 30⋅7	15⋅9 ± 9⋅4	18⋅0 ± 9⋅1^†^
Added sugars	60⋅4 ± 44⋅4	58⋅2 ± 36⋅6	41⋅0 ± 34⋅0	6⋅2 ± 7⋅4^¶^	41⋅6 ± 36⋅7	39⋅8 ± 26⋅5	6⋅3 ± 7⋅7	6⋅1 ± 6⋅8
Total fat	87⋅7 ± 34⋅5	92⋅2 ± 34⋅1	25⋅0 ± 19⋅0	21⋅3 ± 8⋅8^¶^	25⋅0 ± 18⋅1	24⋅8 ± 21⋅0	20⋅9 ± 8⋅6	22⋅4 ± 9⋅4
SFA	31⋅5 ± 13⋅9	32⋅0 ± 11⋅5	9⋅0 ± 7⋅1	7⋅5 ± 3⋅3^¶^	9⋅3 ± 7⋅5	8⋅3 ± 6⋅0	7⋅4 ± 3⋅3	7⋅9 ± 3⋅1
TFA	2⋅9 ± 1⋅6	3⋅1 ± 1⋅7	0⋅93 ± 1⋅1	0⋅68 ± 0⋅40^¶^	0⋅90 ± 1⋅0	1⋅0 ± 1⋅3	0⋅68 ± 0⋅42	0⋅69 ± 0⋅37
Cholesterol	326⋅3 ± 181⋅4	355⋅0 ± 246⋅7	36⋅6 ± 32⋅8	99⋅3 ± 66⋅3^¶^	38⋅6 ± 34⋅9	31⋅8 ± 26⋅5	95⋅9 ± 57⋅7	107⋅7 ± 83⋅5
Dietary fibre	17⋅2 ± 6⋅7	22⋅1 ± 11⋅9*	3⋅3 ± 2⋅7	5⋅1 ± 2⋅8^¶^	3⋅3 ± 2⋅9	3⋅3 ± 2⋅3	4⋅6 ± 2⋅0	6⋅3 ± 4⋅0^†^
Calcium	676⋅6 ± 309⋅6	817⋅4 ± 395⋅2	191⋅1 ± 149⋅2	175⋅5 ± 99⋅1	194⋅8 ± 154⋅8	182⋅0 ± 135⋅1	160⋅6 ± 83⋅5	211⋅8 ± 123⋅0^†^
Iron	14⋅5 ± 5⋅2	17⋅2 ± 6⋅9	3⋅7 ± 2⋅8	3⋅9 ± 1⋅7	3⋅8 ± 2⋅9	3⋅6 ± 2⋅5	3⋅6 ± 1⋅5	4⋅5 ± 2⋅0^†^
Sodium	3175⋅0 ± 1165⋅0	3416⋅1 ± 1102⋅9	910⋅4 ± 574⋅1	778⋅2 ± 327⋅8^¶^	931⋅6 ± 561⋅2	858⋅3 ± 606⋅0	747⋅8 ± 333⋅7	852⋅6 ± 302⋅6^†^
Alcohol	3⋅1 ± 13⋅5	2⋅3 ± 6⋅7	0⋅68 ± 5⋅5	0⋅72 ± 2⋅8	0⋅91 ± 6⋅5	0⋅13 ± 0⋅77	0⋅72 ± 3⋅0	0⋅73 ± 2⋅2

Values are mean ± sd.

NWO, normal-weight obesity; UP, ultra-processed foods; non-UP, non-ultra-processed foods; SFA, saturated fatty acid; TFA, trans fatty acids.

Student's *t* test or Mann–Whitney test (*P*-value <0⋅05).

* Different from NWO group in overall diet.

¶ Different from UP in overall food groups.

† Different from NWO in non-UP food group.

In the final regression model, energy and added sugar from ultra-processed foods were associated with the presence of NWO. Each additional 1 kcal of energy consumption and 1 g of added sugar consumption was associated with a 1⋅02 odds ratio of belonging to the NWO group. Total fat from non-ultra-processed foods was associated with the presence of NWO, with each additional 1 g of total fat intake resulting in a 1⋅09 odds ratio of belonging to the NWO group. Additionally, negative associations were observed between consumption of carbohydrates and total fats from ultra-processed foods and NWO. Each additional 1 g of carbohydrate consumption was associated with a 0⋅93 odds ratio of belonging to the NWO group, and each additional 1 g of total fat consumption was associated with a 0⋅83 odds ratio of belonging to the NWO group (Table 5).

**Table 5.** Associations between independent variables and the presence of normal-weight obesity (*n* 224)

	Estimate	se	OR (95 % CI)	*z*	Pr (>|*z*|)	*P*-value
Intercept	2⋅3729	0⋅57	10⋅72 (3⋅50–32⋅90)	**4⋅15**	**<0⋅001**	**0⋅0000**
UP nutrients						
Energy (kcal)	0⋅01592	0⋅01	1⋅02 (1⋅00–1⋅03)	**2⋅48**	**0⋅013**	**0⋅0100**
Carbohydrate (g)	−0⋅0767	0⋅03	0⋅93 (0⋅88–0⋅98)	**−2⋅66**	**0⋅008**	**0⋅0010**
Added sugars (g)	0⋅0203	0⋅01	1⋅02 (1⋅00–1⋅04)	**2⋅27**	**0⋅023**	**0⋅0100**
Total fat (g)	−0⋅1554	0⋅06	0⋅86 (0⋅76–0⋅97)	**−2⋅51**	**0⋅012**	**0⋅0100**
Non-UP nutrients						
Energy (kcal)	−0⋅0037	0⋅00	1⋅0 (0⋅99–1⋅00)	−1⋅82	0⋅068	0⋅0500
Total fat (g)	0⋅0835	0⋅04	1⋅09 (1⋅01–1⋅18)	**2⋅10**	**0⋅036**	**0⋅0100**
Fibre (g)	−0⋅1336	0⋅07	0⋅87 (0⋅76–1⋅01)	−1⋅81	0⋅071	0⋅0500
Calcium (mg)	−0⋅0039	0⋅00	1⋅0 (0⋅99–1⋅00)	−1⋅94	0⋅052	0⋅0500

Logistic regression with estimate of odds ratio (OR) and the respective confidence interval (95 % CI), with presence/absence of NWO as the outcome. se, standard error; CI, confidence interval; UP, ultra-processed foods.

Variables included in the models were: NWO (yes/no) as the dependent variable; age (years), sex (male/female), energy (kcal) and nutrients [protein (g), carbohydrate (g), total sugars (g), added sugars (g), dietary fibre (g), fat (g), saturated fatty acids (g), trans fatty acids (g), cholesterol (mg), calcium (mg), iron (mg), sodium (mg)], and alcohol (g) from ultra-processed foods; energy and nutrients [(energy (kcal) and nutrients [protein (g), carbohydrate (g), total sugars (g), added sugars (g), dietary fibre (g), fat (g), saturated fatty acids (g), trans fatty acids (g), cholesterol (mg), calcium (mg), iron (mg), sodium (mg)], and alcohol (g) from non-ultra-processed foods, as independent variables.

Bold values indicate significant differences between groups.

## Discussion

This is the first study to analyse food consumption according to the NOVA classification of adults with NWO in Brazil. The results suggest that individuals with NWO, despite presenting with lower energy intake compared with the non-NWO counterparts (*P* = 0⋅030), consumed less fresh food, such as rice, beans and fruits. In addition, a positive association between energy and added sugar intake from ultra-processed foods and a negative association between total fat intake from non-ultra-processed foods with the presence of NWO were observed. These results support the evidence that increased consumption of some dietary components widely found in ultra-processed foods may be associated with harmful outcomes resulting from excess body fat.

In an analysis of a historical series from ten cycles of the National Health and Nutrition Examination Survey (NHANES 1999–2018), US children and adolescents exhibited an increase (from 61⋅4 to 67⋅0 %) in consumption of total energy from ultra-processed foods and a reduction (from 28⋅0 to 23⋅5 %) in the intake of fresh or minimally processed foods^(25)^. Over the last decades, there has been a marked increase in the consumption of ultra-processed foods in several countries, regardless of socioeconomic status. Studies have suggested that this scenario significantly contributes to increased rates of cancer^(26)^, dyslipidaemias^(27)^ and obesity^(28)^ worldwide.

As expected, our study found that ultra-processed foods had higher energy, carbohydrate, fat, sodium, total sugar and added sugars content, but lower protein, cholesterol and dietary fibre compared to non-ultra-processed foods. Although there was no significant difference in ultra-processed food consumption between the NWO and non-NWO groups, there was a positive association between energy and added sugar consumption from ultra-processed foods and the presence of NWO. This finding suggests that a higher intake of energy and added sugar from ultra-processed foods may increase the risk of developing NWO. It is important to note that ultra-processed foods are typically high in calories and added sugars and have been linked to various negative health outcomes. These findings highlight the need to reduce the consumption of ultra-processed foods, and to choose fresh and minimally processed foods instead. Furthermore, the fact that even small changes in energy and added sugar intake can increase the likelihood of developing NWO underscores the importance of making dietary changes to prevent this condition. These findings can have important implications for public health policies aimed at reducing the prevalence of NWO and related diseases.

Regular consumption of ultra-processed foods is associated with an increased risk of mortality from all causes, as well as with impaired intake of whole grains, fruits and vegetables, while consuming leafy vegetables, fruits and other vegetables is associated with lower body weight^(29–31)^. Several mechanisms are involved in the relationship between high consumption of ultra-processed foods and excess adiposity. In general, ultra-processed foods have an ingredient profile considered to be obesogenic compared with fresh and minimally processed foods. These foods tend to be rich in energy, sugars, fats and sodium, while having low levels of dietary fibre, proteins and micronutrients due to the degree of processing^(31,32)^. Moreover, they contain chemical additives that can modify the gut microbiota and contribute to a pro-inflammatory state^(33,34)^. Additionally, these foods are typically stored in plastic containers, which may contain bisphenol A associated with obesity development^(35)^.

Research conducted in Brazil, Chile and Sweden has shown that the purchase of ultra-processed foods has increased significantly in comparison to non-ultra-processed foods^(36–38)^. This trend is not unique to these countries but is rather a global phenomenon that warrants attention. It is crucial to study the impact of consuming ultra-processed foods on the health of individuals and populations, regardless of age and social class. For instance, a recent cohort study involving over 348 000 subjects aged 25 to 70 from nine European countries found that consuming ultra-processed foods was associated with weight gain (0⋅12 kg/5 years, 95 % CI 0⋅09–0⋅15)^(39)^. These results highlight the need to prioritise the consumption of fresh and minimally processed foods to reduce the rates of obesity and other N-NCDs rates.

Previous studies have focused on specific aspects of the association between excess body fat and food consumption. For instance, a study of 4786 Finnish adults found that low consumption of meat and soft drinks was associated with a lower risk of NWO, while high alcohol and sugar intake, as well as low dietary fibre intake, were risk factors^(40)^. Our study's findings are consistent with these results, as individuals with NWO in our sample showed lower dietary fibre intake and higher consumption of processed meats compared to non-NWO subjects. However, no study to our knowledge has evaluated the dietary intake of individuals with NWO according to the NOVA classification. Understanding how different types of processed foods contribute to NWO is crucial to designing effective interventions to prevent and manage this condition.

Our study has several strengths. First, the NOVA classification of foods, despite some criticism, has been widely recommended and it is an important tool for the study of nutrition and public health policies. In addition, the classification of foods into two large subgroups (ultra- and non-ultra-processed) enabled a better understanding of which foods are associated with NWO. Studying individuals with a condition that still requires further investigation (NWO) contributes to an analysis of the consequences of inadequate food consumption, primarily associated with body composition. Finally, the NOVA classification of all foods was carried out by a researcher and rigorously inspected by a second, thus reducing possible bias.

Regarding the study limitations, despite adequate statistical power for inference, the sample size of our study limits the generalisability of our results to other populations. The lack of consensus on cut-off points for NWO classification could also be a limitation, as the results may vary based on the selected %BF values. Nevertheless, we believe that using the cut-off values of >19 % for men and >30 % for women provide a more accurate representation of our studied population, and our findings are consistent with previous NWO research. Additionally, the use of a software based on US nutritional composition tables to assess food consumption in Brazil may introduce potential inaccuracies. However, we standardised the foods, particularly those typical of the local diet, to minimise distortions in the assessment of food consumption.

In conclusion, the overall consumption of ultra-processed foods did not differ between individuals with and without NWO. However, the NWO group consumed more processed meats and had a lower intake of energy and added sugars from non-ultra-processed foods than the non-NWO group. Additionally, significant associations were observed between nutrients from ultra-processed foods and the presence of NWO. While both groups had fresh and minimally processed foods as the basis of their diets, the non-NWO group consumed more non-ultra-processed foods than those with NWO. These findings underscore the importance of the Brazilian dietary guidelines’ golden rule, which advises against consuming ultra-processed foods and recommend making fresh and minimally processed foods the foundation of one's diet.
